# Supplementary material for: Prevalence and Antimicrobial Resistance Profile of Diarrheagenic Escherichia coli from Fomites in Rural Households in South Africa
Source: Antibiotics (Basel). 2023 Aug 21;12(8):1345. doi: 10.3390/antibiotics12081345 (PMC10451885; doi:10.3390/antibiotics12081345)
Supplement: Supplementary file 1 [file antibiotics-12-01345-s001.zip › antibiotics-2531894-supplementary.pdf]

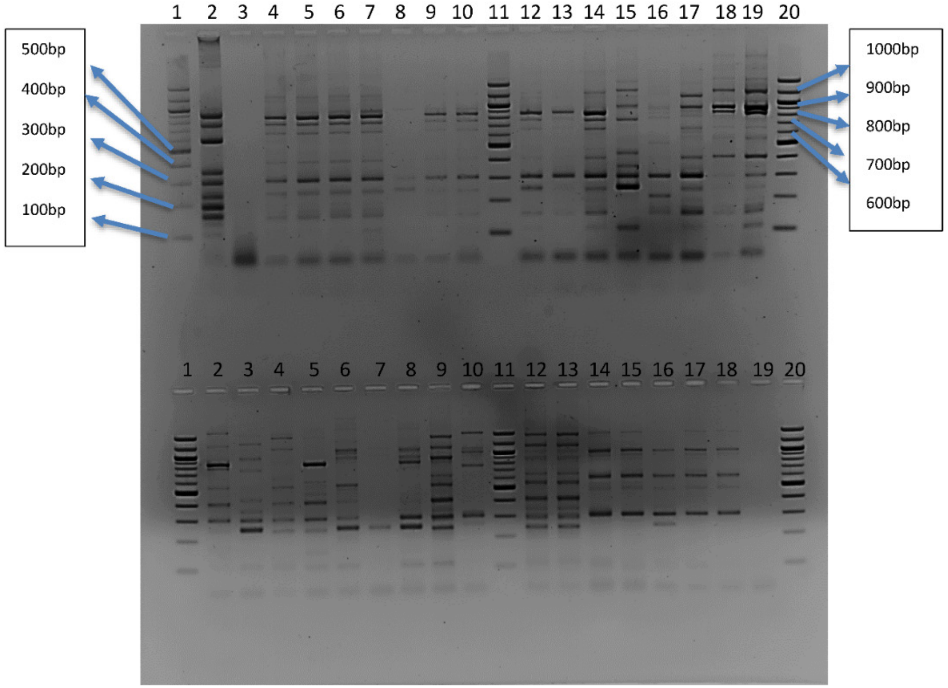

Top wells	Bottom wells
Lane1: 100bp ladder	Lane1: 100bp ladder
Lane2: +ve control	Lane2: H14TH
Lane3: -ve control	Lane3: H16KC
Lane4: H1KC	Lane4: H16TH
Lane5: H1TS	Lane5: H17TH
Lane6: H2KC	Lane6: H18KC
Lane7: H3KC	Lane7: H21TH
Lane8: H3TH	Lane8: H21TS
Lane9: H4KC	Lane9: H22KC
Lane10: H7KC	Lane10: H23KC
Lane11: H7TH	Lane11: ladder
Lane12: H7TS	Lane12: H24KC
Lane13: H8KC	Lane13: H25KC
Lane14: H10KC	Lane14: H26TS
Lane15: H10TH	Lane15: H28KC
Lane16: H10TS	Lane16: H28TH
Lane17: H11KC	Lane17: H28TS
Lane18: H12KC	Lane18: H35KC
Lane19: H14KC	Lane19: xtrection –ve
Lane20: ladder	Lane20: ladder

(**a**) Gel picture A

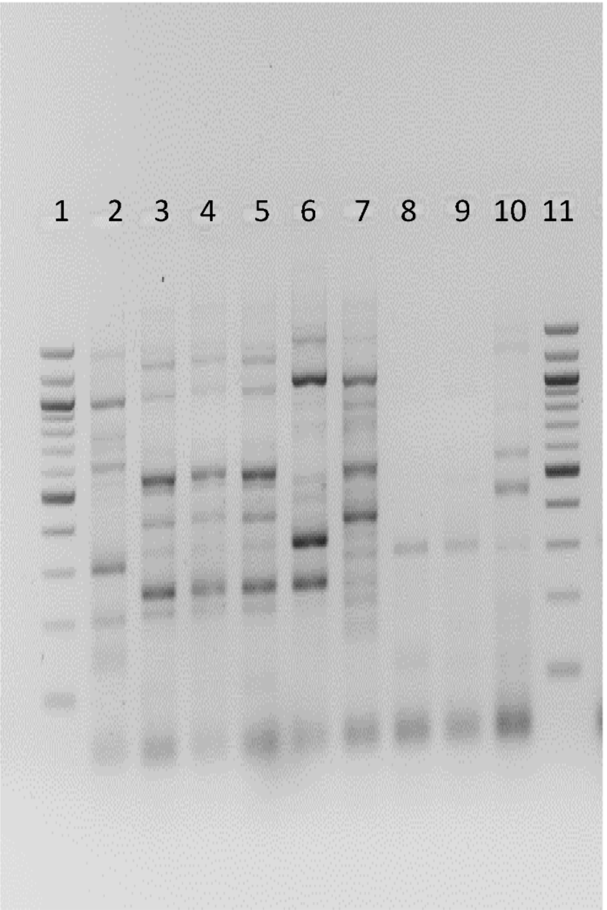

Top wells

Lane1: 100bp ladder
Lane2: +ve control
Lane3: H24TS
Lane4: H32KC
Lane5: H33KC
Lane6: H34KC
Lane7: H30TS
Lane8: H27TH
Lane9: H35TH
Lane10: H35TS
Lane11: ladder

Meaning of the sample code:

H- Household

TS- Toilet seat

TH- Toilet door handle

(**b**) Gel Picture B

Supplementary Figure S1. Depicts gel images A and B, showcasing the multiplex PCR results for detecting different *E. coli* genes on household kitchen cloths and toilet surfaces.
